# Orchestrating Nutrient Homeostasis: RNA-Binding Proteins as Molecular Conductors in Metabolic Disease Pathogenesis

**DOI:** 10.3390/nu17142367

**Published:** 2025-07-19

**Authors:** Siyuan Sun, Xinchun Li, Jianan Zhai, Chenxu Lu, Weiru Yu, Wenhao Wu, Juan Chen

**Affiliations:** Key Laboratory of Precision Nutrition and Food Quality, Department of Nutrition and Health, China Agricultural University, Beijing 100190, China; zb20213311058@cau.edu.cn (S.S.); 18103760496@163.com (X.L.); nn13478543195@163.com (J.Z.); m18637240576@163.com (C.L.); yuweiru0@163.com (W.Y.); s907378510@163.com (W.W.)

**Keywords:** RNA-binding protein, nutrients, obesity, diabetes, LACE-seq

## Abstract

RNA-binding proteins (RBPs) are critical regulators of post-transcriptional processes, playing essential roles in nutrient metabolism and metabolic homeostasis. This literature review explores how RBPs influence the metabolism of glucose, lipid, and amino acid metabolism by controlling processes like mRNA stability and translation regulation. The dysregulation of RBPs, including HuR, PTB, and YTHDF1, is linked to metabolic diseases such as obesity, diabetes, and non-alcoholic fatty liver disease. Advances in techniques like TREX technology and transcriptome analysis have deepened our understanding of RBP functions. Additionally, RBPs show promise as potential biomarkers and targets for new therapies. Future research directions in RBPs could focus on tissue-specific regulation and nutrient–RBP interactions. This could pave the way for more personalized treatments and improved metabolic health.

## 1. Introduction

Nutrients are fundamental to human health, serving as essential components that support bodily functions from the macroscopic level of organ systems down to molecular and genetic processes [1]. Generally, there are six categories of nutrients: carbohydrates, lipids, proteins, vitamins, minerals, and water. Among these, carbohydrates, lipids, and proteins are considered macronutrients, which serve as the energy source of the body; vitamins and minerals are defined as micronutrients, which are needed in smaller amounts but are also vital for life [2]. Beyond energy provision and structural roles [3], nutrients and their metabolites serve as direct modifiers of protein activity [4], potent signaling molecules [5], and regulators of gene expression [6].

**Figure 1:**
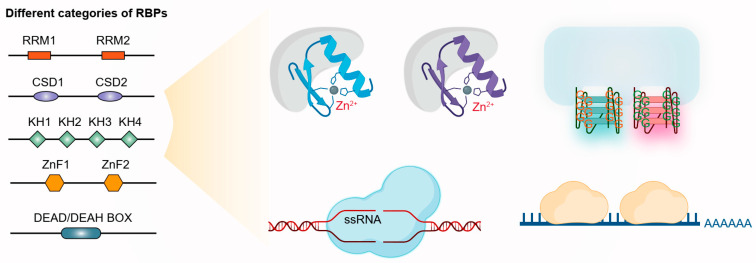
Different RNA-binding proteins categorized by domains and their binding models (RBP: RNA-binding proteins; RRM: RNA recognition motif; CSD: cold shock domains; KH: K homology domain; ZnF: zinc finger motif).

RNA-binding proteins (RBPs) are a diverse group of proteins that play a crucial role in the post-transcriptional regulation (within stages after RNA transcription, including RNA processing, stability, and translation efficiency) of eukaryotic transcriptomes [7]. These proteins interact with RNA molecules in various phases, from transcription to translation, and are involved in a wide range of biological processes. The primary function of RBPs is to bind to specific RNA sequences or structures, thereby influencing RNA stability, localization, translation, and degradation [8]. All of the RBPs have one or more RNA-binding domains (RBDs), which are specific motifs that allow them to interact with RNA (Figure 1). The most common RBDs include zinc finger motifs (ZnFs), RNA recognition motifs (RRMs), cold shock domains (CSDs), K homology (KH) domains, and DEAD/DEAH box helicase domains [9]. The structural and sequence-specific features of RBDs allow RBPs to bind target RNA sequences or structures, such as hairpins, G-quadruplexes, or other secondary structures. This helps RBPs discriminate between different RNA molecules, even in the presence of highly similar sequences, thereby maintaining the fidelity of post-transcriptional regulation.

Understanding the roles of RBPs in nutrients is crucial for unraveling the mechanisms of nutrition regulation and for developing therapeutic strategies in various metabolic diseases. Previous reviews have primarily focused on RBPs in gene regulation or disease contexts. However, very few studies establish a relationship between RBPs and dietary nutrients.

In this review, we explore the intersection of RBPs and nutrient metabolism from an interdisciplinary perspective, focusing on their regulatory roles and functional significance in eukaryotic systems. We aim to summarize the current studies on how RBPs regulate macronutrient and micronutrient metabolism by modulating RNA stability, translation, and degradation, thereby shaping metabolic outputs. Then we elucidate the role RBPs play in the pathogenesis of metabolic disorders and medical treatment at present. We also highlight recent advancements in transcriptomics and technologies, such as Linear Amplification of Complementary DNA Ends and Sequencing (LACE-seq) and Capture RIC-seq (CRIC-seq), which have provided unprecedented insights into the dynamic interplay between RBPs and their target networks. Finally, we demonstrate future research directions, like tissue-specific regulation and nutrient–RBP interactions, which are instrumental to personalized therapies’ efficiency and overall metabolic health improvement.

## 2. RBPs in Nutrient Sensing and Macronutrient Metabolism

### 2.1. Glucose Sensing

RBPs have been reported to transduce the signal of glucose levels in the blood, which promotes downstream reactions such as insulin release and glucagon synthesis.

Poly(C) binding protein 2 (PCBP2) is an RBP that possesses binding specificity for cytosine-rich sequences. The expression of PCBP2 is induced when pancreatic β-cells sense the high glucose level in the blood. β-cells lacking PCBP2 displayed impairments in calcium flux, exocytosis, and insulin secretion [10]. The mRNAs of islet cells were proven to be regulated by the RBP serine/arginine repetitive matrix protein 3 (SRRM3). When the glucose levels are raised, SRRM3 expression also increases. Mice with mutated SRRM3 bear islet cell dysfunction, contributing to hyperinsulinemia hypoglycemia [11].

**Figure 2:**
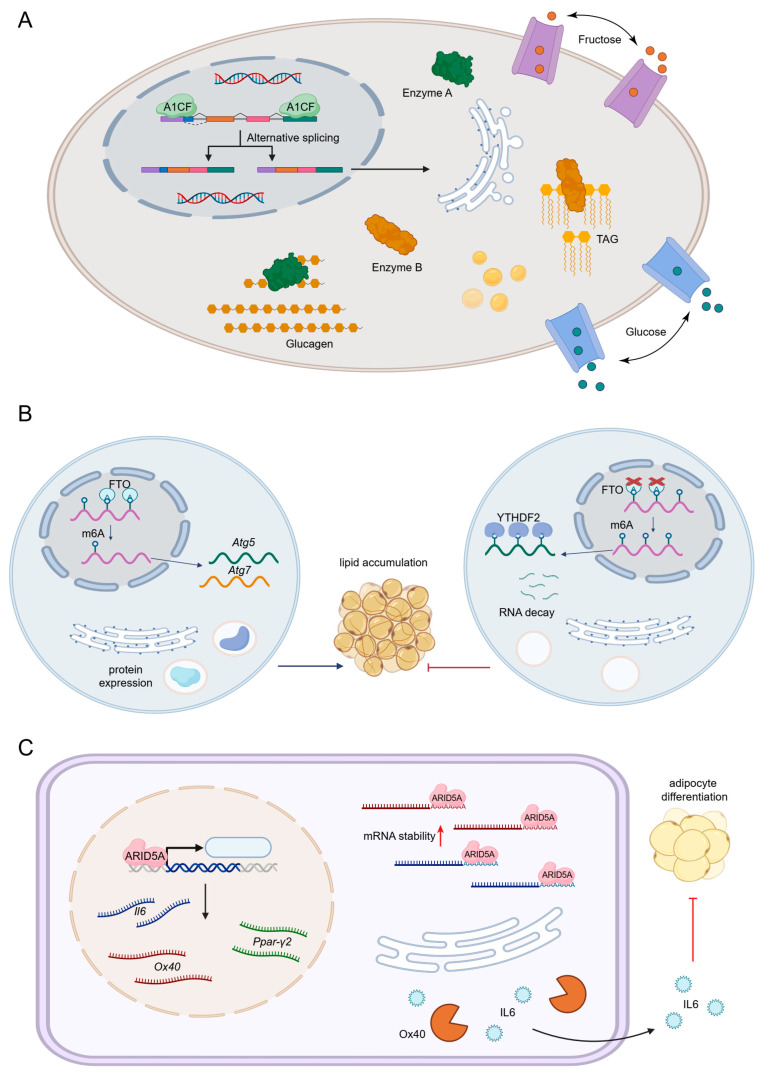
The participation of RBPs in different processes of nutrient metabolism. (**A**) A1CF participates in fructose metabolism through alternative splicing of mRNAs in hepatocytes. (**B**) FTO promotes triglyceride accumulation by m6A demethylation of mRNAs. (**C**) ARID5A regulates adipocyte differentiation via mRNA 3′ untranslated region stabilization. (TAG: triacylglycerol; A1CF: APOBEC1 complementation factor; FTO: fat mass and obesity-associated protein; m6A: N6-methyladenosine; Atg: autophagy-related protein; YTHDF2: YTH N6-methyladenosine RNA-binding protein F2; ARID5A: AT-rich interaction domain 5A; IL6: interleukin 6; Ox40: TNF receptor superfamily member 4; Ppar-γ2: peroxisome proliferators-activated receptor gamma 2).

Apolipoprotein B mRNA editing enzyme catalytic subunit 1 complementation factor (A1CF) is reported to regulate fructose metabolism through alternative splicing in hepatocytes. The target isoforms of A1CF include high-affinity fructose-metabolizing ketohexokinase C and glycerol kinase, both of which are metabolic enzymes participating in hepatic gluconeogenesis (Figure 2A). A1CF-deficient mice demonstrate enhanced glucose tolerance and are resistant to fructose-induced hyperglycemia, liver steatosis, and obesity development [12]. Qiao et al. presented that Sam68 reduces cAMP-response element binding protein-regulated transcription coactivator 2 ubiquitination, a critical transcriptional regulator of gluconeogenesis. Therefore, Sam68 knockouts in hepatocytes have significantly reduced blood glucose levels and the downregulated expression of gluconeogenic genes [13,14].

In hepatocytes, Grb10-interacting GYF protein 2 (GIGYF2) improves Staufen1 stability, which subsequently suppresses PI3K/AKT signaling and induces insulin resistance. When exposed to a high concentration of palmitic acid, the expression of GIGYF2 is upregulated, accompanied by reduced glucose uptake in HepG2 liver cancer cells [15].

Researchers have demonstrated that the formation of protein kinase R (PKR) and TAR RNA-binding protein (TRBP) regulates the phosphorylation of eukaryotic translation initiation factor 2 alpha (eIF2α) [16]. TRBP overexpression aggravated glucose homeostasis impairment in obese mice, but the suppression of hepatic TRBP decreased eIF2α phosphorylation and improved systemic insulin resistance and glucose metabolism. Hence, the PKR-TRBP complex serves as a molecular hub linking metabolic stress to translational regulation, contributing to metabolic homeostasis.

### 2.2. Lipid Signaling and Adipogenesis

In the context of epigenetics, RBPs can influence the expression of genes involved in epigenetic pathways by stabilizing or degrading mRNAs that encode epigenetic enzymes. One well-established mechanism linking RBPs to epigenetic regulation is N6-methyladenosine (m6A), a common form of modification in eukaryotic mRNAs [17]. Fat mass and obesity-associated protein (FTO), a well-known m6A demethylase, promotes autophagosome formation and triglyceride accumulation (Figure 2B). When it is silenced, *Autophagy related 5* (*Atg5*) and *Atg7* transcripts with higher m6A levels were degraded by another RBP, YTH N6-methyladenosine RNA-binding protein F2 (YTHDF2), which alleviates autophagy and adipogenesis [18]. It was found that nicotinamide adenine dinucleotide phosphate (NADP) can directly bind FTO. The NADP/FTO axis increases both FTO activity and the triglyceride fold in 3T3-L1 preadipocytes [19]. Besides, it is reported that human antigen R (HuR) is a major repressor in the process of adipogenesis. HuR binds to and regulates the stability of numerous adipocyte transcripts, including *Insig1*, a key negative regulator of adipogenesis [20]. As a result, the knockout of HuR in the adipose tissue activates the adipogenic gene program, leading to systemic glucose intolerance and insulin resistance. Mynatt et al. demonstrated that the HuR in skeletal muscle modulates a group of genes involved in mitochondrial fatty acid oxidation and oxidative phosphorylation [21]. Another RBP non-POU domain containing octamer binding protein (NONO) was found to accumulate within speckle-like structures in liver cell nuclei. Mice with the removal of NONO have a different mode of energy homeostasis with a high rate of fat oxidation, and they remain lean from infant to adult [22].

It is shown that DEAD-box helicase 1 (DDX1) is phosphorylated in the treatment of palmitate and subsequently releases insulin mRNA, which suppresses insulin translation and elevates glucose level [23]. Sam68, the Src-associated substrate during mitosis of 68 kDa, has demonstrated its vital role in adipogenesis. Through alternative splicing, Sam68 maintains the level of mammalian target of rapamycin (mTOR) transcripts; *Sam68*^−/−^ mice have lower mTORC1/mTORC1 activity and fewer pericytes in white adipose tissue, protecting them against dietary-induced obesity [24].

AT-rich interaction domain 5A (ARID5A) has been identified to stabilize mRNAs by binding to their 3′ untranslated region (UTR) sequences, including Interleukin-6 (IL-6) [25] and Ox40 [26]. Given the connection between IL-6 and ARID5A signaling in adipogenesis, one study highlighted the role of ARID5A in regulating adipogenesis (Figure 2C). IL-6 failed to block adipocyte differentiation in ARID5A-deficient 3T3-L1 cells. Specifically, ARID5A suppressed adipogenesis by inhibiting the transcription of Ppar-γ2, a key regulator of adipocyte differentiation, within the nucleus [27].

### 2.3. Amino Acid Production and Metabolism

There is a group of RBPs that are indispensable in the transport and absorption of amino acids in cells.

Y-box binding protein 3 (YBX3) can be induced by cold stimulation and β-adrenergic signaling in skeletal muscle cells. It stabilizes thermogenesis-related gene transcripts such as the amino acid transporter solute carrier family 3 member 2 (Slc3a2) and Slc1a5 [28], promoting glutamine and branched-chain amino acid (BCAA) uptake [29]. As a result, it supports the proliferation and differentiation of skeletal muscle cells [30]. Musashi2 (MSI2) has been demonstrated as an oncogenic RBP that regulates branched chain amino acid transaminase 1 (BCAT1), a cytosolic aminotransferase for BCAAs. MSI2 contributes to the aberrant activation of BCAT1 in chronic myeloid leukemia cells, which uplifts the BCAA production [31].

m6A and its associated RBPs have been linked to pathological processes, including hematopoiesis impairment and metabolic disorders. RNA-binding motif protein 15 (RBM15) has been reported as an m6A methyltransferase, which directly binds to target serine and glycine metabolic genes, including PHGDH, PSAT1, PSPH, and SHMT2 [32]. Therefore, RBM15 increases the serine and glycine flux in cells. In a benzene-induced hematopoietic damage mouse model, insulin-like growth factor 2 mRNA-binding protein 1 (IGF2BP1) was found to be a critical RBP in BCAT1 and carnitine palmitoyltransferase 1A (CPT1A) metabolic enzymes, which improve hematopoietic injury via BCAA metabolism and fatty acid oxidation [33]. Moreover, IGF2BP2 has been reported to be involved in acute myeloid leukemia development by regulating a group of genes, including MYC, GPT2, and SLC1A5. By m6A modification, IGF2BP2 activates the gene expression in the glutamine metabolism pathways [34].

Angiogenin (ANG) is an RNase-A-family RBP known for promoting endothelial cell proliferation and angiogenesis [35]. Recent research has revealed a novel, non-vascular role of ANG in neuronal metabolism. Specifically, in glutamatergic neurons of the adult brain, ANG undergoes dephosphorylation-induced translocation from the nucleus to the cytoplasm. Once in the cytoplasm, ANG interacts with tRNAs and catalyzes the precise cleavage of nucleus-encoded tRNA^Glu^, generating a specific transfer-RNA-derived small RNA known as Glu-5′ tsRNA-CTC. This tsRNA exerts downstream effects by binding to leucyl-tRNA synthetase 2 (LARS2), an essential mitochondrial enzyme responsible for aminoacylating mitochondrial tRNA^Leu^ (mt-tRNA^Leu^) with leucine. Upon complex formation, Glu-5′ tsRNA-CTC and LARS2 translocate to the mitochondria, where the interaction inhibits the aminoacylation activity of LARS2. Consequently, this blockade disrupts the mitochondrial translation machinery by impairing the synthesis of proteins [36].

## 3. Interactive Influence Between Micronutrients and RBPs

### 3.1. Vitamins

Retinoic acid (RA), the derivative of vitamin A, is considered a chemopreventive agent for breast cancer. RA treatment recovers the translation level of HOXA5, which controls cell growth. This process needs the participation of HuR, which binds to 3′ UTR of HOXA5 mRNA and increases its stability [37].

Insulin-like growth factor-2 mRNA-binding protein 3 (IGF2BP3) has been implicated in inflammatory responses. In a high glucose (HG)-induced retinal pigment epithelial (RPE) cell model, ascorbic acid (vitamin C) was shown to alleviate HG-induced RPE cell damage by inhibiting the NF-κB signaling pathway through regulation of the MALAT1/IGF2BP3 axis [38]. In one study, dietary vitamin C shows therapeutic potential in Fragile X Syndrome (FX) by reducing DNA methylation at the X-linked Fragile X Messenger Ribonucleoprotein 1 (FMR1) gene locus, thereby restoring its expression in FX-induced pluripotent stem cells (iPSCs) and cerebral organoids [39]. Given that certain RNA-binding proteins possess methyltransferase activity, further research may focus on which RNA-binding protein participates in the FMR1 regulation in the presence of vitamin C.

As for cobalamin (vitamin B12), it has been reported that patients with inborn defects in cobalamin metabolism have altered shuttling and splicing of the mRNA repertoire, caused by the dysfunction of RBPs, including ELAVL1/HuR, HnRNPA1, and RBM10 [40].

Researchers have demonstrated that the levels of the vitamin D receptor (VDR) in the intestinal mucosa decreased significantly in mice with the removal of HuR. In intestinal epithelial cells (IECs), Vdr mRNA is modulated by HuR, which binds to the 3′-untranslated region (UTR) and thus enhances VDR translation [41]. Another study about ovarian cancer has shown the synergistic effect of VDR and RBP. It is reported that the expression of lncRNA TOPORS antisense RNA 1 (TOPORS-AS1) is positively correlated with the overall survival of patients. The vitamin D receptor (VDR) upregulated the expression of TOPORS-AS1 while hnRNPA2B1 (heterogeneous nuclear ribonucleoprotein A2B1) directly interacted with TOPORS-AS1, both of which elicit the inhibition of β-catenin and suppress the proliferation of ovarian cancer cells [42].

### 3.2. Iron Homeostasis

IRP1 is an RNA-binding protein that regulates iron homeostasis post-transcriptionally [43]. Researchers have found that it binds to iron-responsive elements (IREs), which are stem-loop structures in the 5′ UTR of ferritin mRNA and the 3′ UTR of transferrin receptor mRNA. IRP1 binds to 5′ UTR IREs to inhibit translation in iron-deficient cells, while it stabilizes the mRNA and prevents degradation when binding to 3′ UTR IREs [44]. In this way, IRP1 maintains appropriate intracellular iron levels.

HAMP encodes hepcidin, another key regulator of systemic iron homeostasis. The 3′ UTR of HAMP mRNA is enriched with canonical cytoplasmic polyadenylation element (CPE) motifs [45], which have been identified as binding sites for the RNA-binding protein CPEB4 [46]. Notably, CPEB4 plays a critical role in modulating cancer cell sensitivity to ferroptosis, a form of regulated cell death driven by iron-dependent lipid peroxidation. Loss of CPEB4 function leads to reduced hepcidin expression, resulting in the upregulation of ferroportin and decreased intracellular iron levels. These changes collectively suppress ferroptosis, highlighting the significance of the CPEB4-hepcidin axis in iron metabolism and cancer cell death pathways [47].

YTH N6-methyladenosine RNA-binding protein F1 (YTHDF1) and YTHDF2 play an essential role in binding ferritin heavy chain 1 (FTH1) mRNA to improve its stability and translation. The knockdown of FTO, the m6A demethylase, leads to increased m6A modification on FTH1 mRNA. This impairs recognition by the m6A reader proteins YTHDF1 and YTHDF2, resulting in reduced intracellular levels of FTH1. Without sufficient FTH1 to bind and store Fe^2+^, spermatogenic cells undergo ferroptosis [48].

## 4. Dysregulation of RBPs and Metabolic Diseases

### 4.1. Obesity and Insulin Resistance

Obesity, recognized as a globally prevalent metabolic disorder, arises from dysregulated energy homeostasis and lipid metabolism [49]. Emerging evidence has elucidated the mechanistic role of RBPs in modulating obesity-associated pathways through post-transcriptional regulation [50,51]. In general, RBPs can directly govern the expression of genes critical for adipogenesis, thermogenic energy expenditure, and insulin signaling cascades.

RBPs contribute to obesity through regulating genes related to thermogenesis. The RNA-binding protein quaking (QKI) is a vital regulator of the metabolic homeostasis of adipose tissue. Studies have shown that QKI-deficient mice exhibit resistance to high-fat diet (HFD)-induced obesity, primarily due to enhanced energy expenditure. Specifically, QKI depletion promotes thermogenic activity by increasing energy dissipation in brown adipose tissue and stimulating the browning of subcutaneous white adipose tissue. Functionally, QKI suppresses energy consumption in adipose tissue by destabilizing mRNAs and impairing their nuclear export and translation—particularly those encoding key thermogenic regulators UCP1 (uncoupling protein 1) and PGC1α (peroxisome proliferator-activated receptor gamma coactivator 1-alpha) [52]. Dai et al. found that *Imp2*^−/−^ mice exhibit marked resistance to diet-induced obesity and hepatic steatosis. Adipocytes in the mice model with Imp knockout demonstrate significantly enhanced uncoupled oxygen consumption, indicative of elevated thermogenic activity. IMP2 has been shown to bind to the mRNAs encoding UCP1 and various mitochondrial components, where it acts as a translational repressor. In the absence of IMP2, these target mRNAs display increased translational efficiency, contributing to enhanced mitochondrial function and energy expenditure [53].

Another role of RNA-binding proteins (RBPs) is their active involvement in the regulation of adipogenesis. It is reported that paraspeckle component 1 (PSPC1) promotes obesity by facilitating adipogenesis and enhancing fat storage. As an RNA-binding protein, it regulates key adipogenic RNAs, such as EBF1, and collaborates with DEAD-box helicase 3 X-linked (DDX3X) to export these RNAs during cell differentiation. This process increases adipocyte formation and lipid accumulation. Furthermore, in mice lacking PSPC1 specifically in adipose tissue, there is reduced fat mass and lipid storage, leading to resistance to diet-induced obesity and insulin resistance. This protective effect stems from a compensatory increase in energy expenditure [54]. One clinical trial found that Paral1, a long intergenic noncoding RNA (lincRNA), is a novel biomarker of obesity-related dysregulation of adipose tissue function [55]. Paral1 acts as an obesity-sensitive regulator of adipocyte differentiation and metabolic function. Paral1 exerts its effect through interactions with RBM14, a paraspeckle-associated RNA-binding protein with hnRNP-like features. It promotes adipogenesis by acting as a coactivator of peroxisome proliferator-activated receptor gamma (PPARγ), a key transcription factor governing adipocyte development. One study showcased the pivotal role of YBX1 in driving fat accumulation, a core feature of obesity. YBX1 is a 5-methylcytosine (m5C)-binding protein, and its deficiency inhibits adipocyte differentiation in murine models while the overexpression of YBX1 in white adipose tissue increases ULK1/ULK2 expression and adipogenesis by promoting autophagy controlled by ULK1 and ULK2. Mechanistically, YBX1 directly targets and stabilizes m5C-modified Ulk1 mRNA, thereby upregulating ULK1 expression. Simultaneously, YBX1 acts as a transcription factor to promote Ulk2 transcription and expression. Collectively, YBX1 controls autophagy and adipogenesis, positioning it as a key regulator for obesity driven by adipose tissue expansion [56].

Recently, researchers developed adipose-specific zinc finger protein 36 knockout (ZFP36 KO) mice and exposed them to a 16-week high-fat diet (HFD). They elucidated that ZFP36 deficiency-driven obesity manifested through white adipose tissue adipocyte hypertrophy, mediated by the reduced expression of lipid metabolism regulators PLIN1, ATGL, and HSL. Mechanistically, ZFP36 directly suppressed RNF128 expression through dual inhibition of mRNA stability and translation, which enhanced Sirt1-mediated metabolic homeostasis. Their findings establish adipose ZFP36 as a critical modulator of diet-induced obesity in the ZFP36/RNF128/Sirt1 signaling axis [57]. Meng et al. demonstrated that hnRNPA2B1 (A2B1) mRNA levels are elevated in the epididymal white adipose tissue (eWAT) and spleen of high-fat diet (HFD)-induced obese mice, as well as in colon tissues during inflammation. Notably, mice with a haploinsufficiency of A2B1 exhibit resistance to HFD-induced obesity, characterized by reduced white adipose tissue expansion. Mechanistically, A2B1 enhanced the RNA stability of pro-inflammatory genes, including TNF-α, IL-6, and IL-1β, for the intensified polarization of pro-inflammatory M1 macrophages in eWAT [58].

As for hormones, PTB (polypyrimidine tract-binding protein) plays a critical role in the post-transcriptional regulation of adiponectin receptor 1 (AdipoR1), which mediates adiponectin’s metabolic benefits in muscle and liver. PTB binds to the 3′ UTR of AdipoR1 mRNA and cooperates with microRNA-221 (miR-221) to suppress its translation. In mouse models of obesity, both PTB and miR-221 are upregulated in muscle and liver tissues, contributing to the pathological decline in AdipoR1 protein. Reduced AdipoR1 exacerbates insulin resistance by diminishing adiponectin’s ability to enhance glucose uptake and lipid metabolism. Conversely, the depletion of PTB or miR-221 restores AdipoR1 translation, suggesting that targeting PTB could rescue adiponectin signaling and ameliorate metabolic dysfunction in obesity [59]. ZFP36L1 is an RBP that binds to AU-rich elements in the 3′ UTRs of mRNAs for their degradation [60], which was identified as a target gene of the bile-acid-activated nuclear receptor called farnesoid X receptor (FXR). It participates in bile acid metabolism and lipid homeostasis. Experimental studies demonstrate that modulating ZFP36L1 function reciprocally controls Cyp7a1 mRNA stability and bile acid levels in vivo. Mice lacking hepatic ZFP36L1 exhibit resistance to diet-induced obesity and hepatosteatosis. These findings establish ZFP36L1 as a key metabolic regulator linking FXR signaling to obesity and fatty liver disease through bile-acid-dependent pathways [61].

### 4.2. Non-Alcoholic Fatty Liver Disease

Non-alcoholic fatty liver disease (NAFLD), the hepatic manifestation of metabolic syndrome, is characterized by impaired lipid metabolism, fat accumulation, and lipid droplet formation. NALFD has the risk of developing liver fibrosis, cirrhosis, or even hepatocellular carcinoma [62]. RBPs are associated with the pathogenesis of NAFLD for their involvement in triglyceride metabolism.

The RNA-binding protein HuR (ELAVL1), which is popular among researchers, has been proven to take an active part in the development of NALFD [63]. In the cytoplasm, HuR binds to AU-rich elements (AREs) within the 3′ UTR of target mRNAs via its RNA recognition motifs (RRMs), thereby enhancing mRNA stability by preventing degradation and deadenylation. RRM1 and RRM2 typically recognize A/U-rich or U-rich sequences, while RRM3 specifically binds to U-rich regions [64]. Liu and his colleagues have demonstrated that HuR acts as a dual regulator of intestinal triglyceride (TAG) synthesis by modulating the post-transcriptional stability of Dgat2 mRNA and the splicing efficiency of Mgat2 pre-mRNA. Specifically, HuR binds to the 3′ untranslated region (UTR) of Dgat2 mRNA to enhance its stability, while simultaneously interacting with intron 1 of Mgat2 pre-mRNA to facilitate its processing into mature mRNA. These complementary mechanisms elevate the expression of DGAT2 and MGAT2, key enzymes in intestinal TAG synthesis, thereby promoting dietary fat absorption. Consequently, HuR deficiency mitigates high-fat diet (HFD)-induced non-alcoholic fatty liver disease (NAFLD) and obesity [65]. HuR also plays a protective role against metabolic syndrome-related diseases, primarily by regulating cholesterol transport. ABCB1 is a key mediator of reverse cholesterol transport, facilitating cholesterol efflux and HDL formation. It has been shown that HuR enhances ABCA1 expression by stabilizing its mRNA through binding to the 3′ UTR. Silencing HuR can result in decreased ABCA1 levels and reduced cholesterol efflux to APOA1 in hepatocytes [66].

One study reveals that QKI 5, a critical RNA-binding protein, regulates hepatic triglyceride synthesis in a non-alcoholic fatty liver disease (NAFLD) model. QKI 5 upregulated the expression of PPARα through post-transcriptional regulation, which further inhibited TAG synthesis and NAFLD progression [67]. Insulin-like growth factor 2 mRNA-binding protein 2 (IMP2) is involved in hepatic triglyceride metabolism. Mice with liver-specific IMP2 overexpression are prone to steatosis. Hepatocyte-specific IMP2 knockout exacerbates diet-induced fatty liver by impairing fatty acid oxidation, resulting from the increased degradation of IMP2 target mRNAs, PPARα, and CPT1A. These findings suggest that IMP2 deficiency is attributed to the reduced adiposity [68].

### 4.3. Diabetes

For mammals, the primary cause of diabetes is the dysfunction or loss of pancreatic β-cells, which are specialized cells located in the islets of Langerhans within the pancreas. These β-cells are responsible for secreting insulin, a hormone essential for maintaining blood glucose homeostasis [69]. However, when these cells become dysfunctional—either through impaired insulin secretion, loss of β-cell mass, or both—this regulation fails, resulting in chronic hyperglycemia, the hallmark of diabetes mellitus [70]. Clinical observations have reported significantly altered expression levels of multiple proteins in patients with diabetes [71,72]. Since the dysregulation of RBPs can induce functional abnormalities in their downstream target genes, these protein-level disturbances correlate with disease progression.

There are two major mechanisms through which RBPs are involved in diabetes. First, RBPs can affect the process of insulin secretion. Stoll et al. demonstrated that there are numerous circular RNAs (circRNAs) that are expressed in pancreatic islets, and many of them regulate β-cell activities. The researchers further found an interaction between the RBP, trans-activator regulatory DNA-binding protein 43 (TDP-43), and intron lariats of ci-Ins2. The abundance of this circularized intron is decreased in the islets of both rodent models of diabetes and individuals with type 2 diabetes. Since ci-Ins2 promotes insulin release, the existence of TDP-43 is crucial for β-cells to secrete insulin [73]. CUG-binding protein 1 (CUGBP1) is a multifunctional RBP that regulates various aspects of RNA metabolism, preferentially binding to GU-rich elements [74]. CUGBP1 is expressed in rodent islets and β-cell lines and is notably upregulated in the islets of diabetic mice [75]. It is demonstrated that pancreas-specific overexpression of CUGBP1 impairs glucose tolerance, as evidenced by reduced plasma insulin levels following glucose challenge and delayed glucose clearance. Conversely, CUGBP1 knockdown improves insulin secretion and glucose handling, indicating a deleterious role for CUGBP1 in glucose homeostasis. Mechanistically, CUGBP1 overexpression attenuates glucose- and GLP-1-stimulated insulin secretion, which correlates with a marked reduction in intracellular cAMP levels—a key second messenger in insulin exocytosis. CUGBP1 specifically increases the expression of PDE3B, an enzyme known to inhibit insulin secretion by lowering cAMP. This occurs through direct binding of CUGBP1 to an ATTTGTT motif in the 3′ UTR of PDE3B mRNA, thereby stabilizing the transcript and promoting its accumulation. Notably, both CUGBP1 and PDE3B are also overexpressed in diabetic islets, further supporting a pathological link between the CUGBP1–PDE3B axis and β-cell dysfunction. Poly(A)-specific ribonuclease (PARN) is widely recognized for its role in regulating mRNA stability through poly(A) tail shortening [76]. Recent findings have reported its involvement in pancreatic β-cell physiology [77]. From β-cell-specific PARN knockout mice, the absence of PARN leads to a marked reduction in glucose-stimulated insulin secretion (GSIS). Transcriptomic profiling of PARN-deficient β-cells reveals the misregulation of genes essential for insulin secretion. In detail, PARN interacts with the RNA-binding protein polypyrimidine tract binding protein 1 (PTBP1) to regulate the stability of key transcripts, including Slc30a8, which encodes a zinc transporter vital for insulin crystallization, and Chst3, which is implicated in vesicle trafficking and secretion. Disruption of either PARN or PTBP1 compromises the stability of these mRNAs, leading to defects in insulin maturation and exocytosis. Therefore, PARN serves as a crucial regulator of β-cell secretory capacity, and targeting the PARN–PTBP1–mRNA axis may offer novel therapeutic avenues for improving insulin secretion in diabetes.

In addition, RBPs contribute to the maintenance of pancreatic cell homeostasis and the dynamic modulation of the microenvironment. The RNA-binding protein HuD plays a vital role in post-transcriptional gene regulation and has been implicated in the pathogenesis of various diseases, including diabetes [78,79]. Hong et al. found that in both db/db diabetic mice and HuD knockout models, mitochondrial fragmentation was increased, indicating impaired mitochondrial fusion, a main phenotype of β-cell failure in diabetes [80]. HuD binds to the 3′ UTR of mitofusin 2 (Mfn2) mRNA, a key mediator of mitochondrial fusion, and positively regulates its expression. In vitro, HuD depletion in mouse insulinoma βTC6 cells leads to a higher proportion of cells with fragmented mitochondria, accompanied by decreased mitochondrial membrane potential and reduced ATP production, reflecting diminished mitochondrial function. Notably, the ectopic expression of Mfn2 in HuD-depleted cells was sufficient to rescue mitochondrial morphology and function, confirming that HuD exerts its protective role via the HuD–Mfn2 axis. Another group of researchers identified endostatin and Serpin E1—two proteins with well-known anti-angiogenic properties—as HuD-regulated factors by using a cytokine array in mouse insulinoma βTC6 cells [81]. Specifically, HuD knockdown led to elevated levels of Col18a1 (the precursor of endostatin) and Serpin E1 by binding to their 3′ UTRs and repressing their translation. Functional analyses using co-culture systems of βTC6 cells and islet endothelial MS1 cells revealed that HuD downregulation impaired endothelial cell growth and migration, indicating a key role for HuD in mediating β-cell–endothelial cell crosstalk. Moreover, ectopic expressions of HuD in db/db diabetic mice reduced Col18a1 and Serpin E1 expression while enhancing the expression of vascular markers in islet tissue, suggesting a protective role in maintaining islet vascular homeostasis. A genome-wide analysis of circRNA expression in peripheral blood mononuclear cells (PBMCs) from type 1 diabetes mellitus (T1DM) patients identified circular protein phosphatase 1F (circPPM1F) as significantly upregulated compared to healthy controls [82]. In streptozotocin (STZ)-induced diabetic mice, circPPM1F overexpression worsened pancreatic damage through its activation of M1 macrophages. Functionally, circPPM1F promotes lipopolysaccharide (LPS)-induced M1 macrophage activation by enhancing NF-κB signaling, a key inflammatory pathway implicated in β-cell destruction in T1DM. Mechanistically, circPPM1F acts as a molecular sponge for HuR, competitively binding to it and thereby impairing HuR-mediated translation of PPM1F—a negative regulator of NF-κB signaling. By inhibiting PPM1F translation, circPPM1F removes a critical brake on inflammatory activation, thereby exacerbating macrophage-driven pancreatic injury. The elevated expression of circPPM1F is maintained by the RBPs—EIF4A3 and FUS, highlighting a complex RBP-regulated network in T1DM pathogenesis.

## 5. Research Methods and Technological Advances

### 5.1. CRISPR/Cas9 Library Screening: Identifying Metabolism-Related RBPs

**Figure 3:**
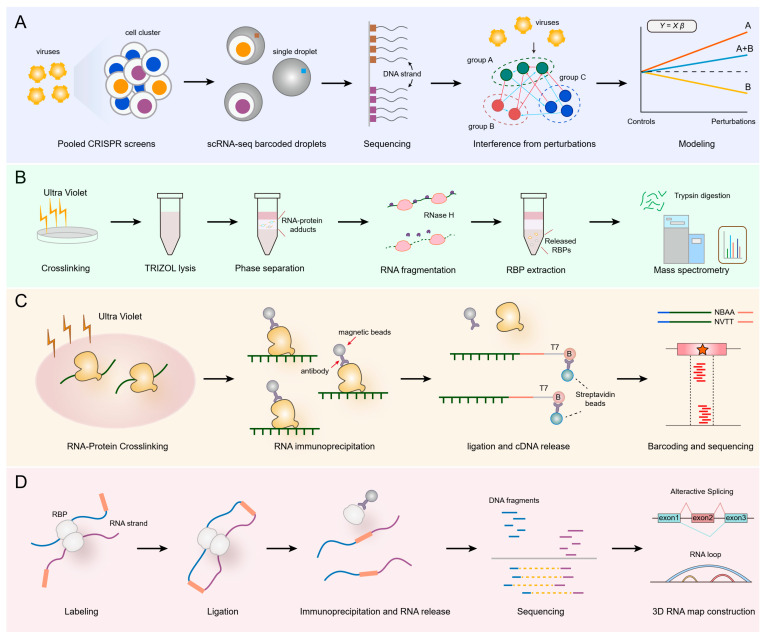
Advanced technologies in discovering the characteristics of RBPs. (**A**) Perturb Sequencing (Perturb-seq). (**B**) Targeted RNase H-mediated extraction (TREX) technology. (**C**) Linear Amplification of Complementary DNA Ends and Sequencing (LACE-seq). (**D**) Captured RNA in situ conformation sequencing (CRIC-seq).

Clustered regularly interspaced short palindromic repeats and their associated protein 9 (CRISPR/Cas9)-based functional genomics have emerged as a powerful tool for uncovering metabolism-related RBPs. Pooled knockout screens using sgRNA libraries targeting hundreds to thousands of RBP genes have pinpointed factors like INTS3, whose loss induces apoptosis in colorectal cancer cells [83]. Patrick et al. demonstrated a reporter-based RNA-linked CRISPR (ReLiC), which embeds barcoded RNA reporters at a defined genomic locus to directly assay diverse RNA metabolic events—splicing, translation, and decay—upon gene knockout [84]. Single-cell CRISPR screens such as Perturb-seq (Figure 3A) integrate pooled CRISPR perturbations with single-cell RNA sequencing to map transcriptome-wide effects of RBP loss on metabolic gene programs [85]. Amit et al. adopted focused sub-genomic CRISPR screens and revealed putative RBP vulnerability in MLL-translocated B-ALL [86]. CRISPR interference/activation (CRISPRi/a) platforms modulate RBP expression without double-strand breaks, facilitating the interrogation of essential or dosage-sensitive RBPs in metabolism [87]. Overall, these advanced CRISPR/Cas9 techniques enable more precise validation of the specific functions of RBPs.

### 5.2. TREX Technology: Mapping RNA–Protein Interaction Networks in Live Cells

TREX, with the full name of targeted RNase H-mediated extraction of crosslinked RBPs, is a revolutionary technique (Figure 3B). It is designed to identify RBPs that interact with specific RNA regions in living cells under physiological conditions [88]. Unlike existing methods, this approach combines RNase H-mediated cleavage with crosslinking to isolate proteins that directly bind to defined RNA regions. TREX uses two-step phase separation: the first one is after UV-C crosslinking, which extracts all the RNA–protein adducts; the second one is after antisense DNA oligonucleotides hybridize to the target RNA, which releases the RBPs for further quantitative mass spectrometry (MS) analysis. Since it enables the precise removal of unrelated RNA–protein adducts and the enrichment of RBPs binding to the RNA sequence of interest, it offers unparalleled sensitivity and specificity compared to other techniques. For example, TREX detects up to 27 direct U1 snRNA interactors, in contrast to the current reported 10 U1 snRNP components [89,90]. By systematically mapping the interactome of 45S rRNA and NORAD long noncoding RNA, TREX has shown its effectiveness in identifying functional interactors of a specific RNA region under endogenous settings. Hence, TREX could serve as a powerful RNA-centric methodology for studying positional RNA–protein interactions across diverse cell types, providing researchers with a robust tool to explore the nutrient conversion and regulation in living systems.

### 5.3. LACE-Seq

LACE-seq, which is short for Linear Amplification of Complementary DNA Ends and Sequencing, is an advanced technique developed to profile RBP target sites across the transcriptome (Figure 3C), particularly in low-input samples such as oocytes [91] and spermatocytes [92]. This method addresses the limitations of traditional approaches like crosslinking-immunoprecipitation and high-throughput sequencing (CLIP-seq), which often require large amounts of starting material and may not be suitable for studying rare cell populations [93].

This technique has been widely applied to study various RBPs in mouse models. For instance, Su et al. [94] utilized LACE-seq to investigate the binding sites of RBPs such as Argonaute 2 (Ago2), Mili, and Ddx4. Their findings revealed that Ago2 interacts with endogenous small interfering RNAs (endo-siRNAs) to globally repress mRNA translation in oocytes. Furthermore, the Ago2-endo-siRNA complexes were found to fine-tune the transcriptome by slicing long terminal repeat retrotransposon-derived chimeric transcripts. In another study, LACE-seq was employed to analyze the binding patterns of eukaryotic translation initiation factor 4E family member 1B (eIF4E1B), a translation initiation factor, in mouse oocytes [95]. The results indicated that eIF4E1B preferentially binds to the 5′ UTRs of target mRNAs, particularly those containing GC-rich motifs, thereby selectively activating the translation of maternal mRNAs during the oocyte-to-embryo transition. Lei et al. utilized LACE-seq to demonstrate that SRSF1 plays an essential role in spermatogenesis and male fertility by regulating alternative splicing (AS). Male mice with germ cell-specific deletion of Srsf1 bear complete infertility, which has the abnormal AS of genes involved in the spermatogenesis process, such as Stra8, Dazl, and Dmc [96].

### 5.4. CRIC-Seq

CRIC-seq is an innovative technique designed to map RNA–RNA interactions mediated by specific RNA-binding proteins (RBPs) within living cells, offering profound insights into post-transcriptional gene regulation [97]. Building upon the foundation of RNA in situ conformation sequencing (RIC-seq), CRIC-seq introduces an immunoprecipitation step to isolate RNA–RNA contacts associated with particular RBPs (Figure 3D), thereby enhancing the specificity and depth of interaction profiling [98]. While earlier techniques like hiCLIP primarily detected RNA–RNA interactions based on sequence complementarity, CRIC-seq’s use of formaldehyde crosslinking allows for the capture of protein-mediated interactions, which allows one to detect long-range RNA interactions and provides a more comprehensive view of the RBP-associated RNA interactome [99].

CRIC-seq is a useful tool for elucidating the role of RBPs in alternative splicing. For example, studies utilizing CRIC-seq to investigate PTBP1, SRSF1, and hnRNPA1 in HeLa cells revealed that PTBP1-associated RNA loops within individual introns tend to promote exon inclusion by facilitating asymmetric intron removal. Conversely, loops spanning across exons often repress splicing. These findings underscore the significance of the spatial positioning of RBP-mediated RNA loops in determining splicing outcomes [100].

## 6. Clinical Translation and Future Directions

### 6.1. Therapeutic Strategies Targeting RBPs for Metabolic Diseases

Due to the dysregulation of RBPs in metabolic disorders such as obesity, NAFLD, and diabetes, therapeutic strategies aimed at modulating RBP activity are gaining traction.

Zhuang and his colleagues highlight niclosamide (NCS), an HuR inhibitor, as a potential therapeutic agent for Diabetic Nephropathy (DN) [101]. NCS treatment in db/db mice reduced hyperglycemia (lowered blood glucose and HbA1c) without affecting body weight, effectively halting DN progression. HuR activation upregulated Wisp1, a downstream effector of Wnt1 signaling, in diabetic kidneys, while NCS treatment restored Wisp1 to normal levels. HuR inhibition subsequently attenuated renal inflammation by suppressing NF-κBp65, TNF-α, and MCP-1 expression.

Wang et al. reveal that endostatin inhibits adipogenesis and dietary-induced obesity. Endostatin exerts its anti-adipogenic effects by interacting with the RNA-binding protein Sam68 within the nuclei of preadipocytes. This interaction competitively disrupts Sam68’s binding to intron 5 of the mammalian target of the rapamycin (mTOR) transcript, resulting in aberrant mTOR mRNA processing. As a consequence, mTOR expression is reduced, leading to diminished activity of the mTOR complex 1 (mTORC1) pathway and impaired adipogenesis [102].

One interesting study showed that BCAA supplementation can inhibit adipogenesis and obesity [103]. It first reduces the levels of nicotinamide adenine dinucleotide phosphate (NADPH) in adipose tissue with decreased expression of FTO. This reduction in FTO activity increases m6A methylation on Ccna2 and Cdk2 mRNAs, which are subsequently recognized by the m6A reader protein YTHDF2. YTHDF2 quickens mRNA decay and reduces the protein levels of CDK2 and CCNA2. This NADPH-FTO- m6A regulatory axis offers a new option for alleviating obesity.

Another study reported that metformin prevents obesity in a similar way. Metformin can significantly suppress FTO expression, leading to elevated m6A methylation of Ccnd1 and Cdk2 mRNAs. This modification promotes their recognition by the m6A reader protein YTHDF2, resulting in reduced protein levels of CCND1 and CDK2 [104].

A small molecule, ABX300, has been identified as a potential therapeutic agent capable of mitigating diet-induced obesity by modulating LMNA isoform expression through the regulation of serine/arginine-rich splicing factor 1 (SRSF1) in HFD-fed mice [105]. SRSF1 is a key regulator of pre-mRNA splicing that causes abnormal gene expression through post-translational regulation [106]. Treatment with ABX300 was shown to reverse HFD-induced aberrant splicing of LMNA through the inhibition of SRSF1 activity. Beyond its splicing modulatory effects, ABX300 also altered the metabolic rate and energy expenditure in HFD-fed mice by upregulating genes involved in fat utilization and lipid catabolism, without decreasing the lean weight of mice.

Platania et al. identified two indole-containing derivatives, VP12/14 and VP12/110, as modulators of HuR (ELAVL1) expression with therapeutic potential in the context of diabetic retinopath [107]. In human retinal endothelial cells (HRECs) exposed to high glucose (HG) conditions, both compounds significantly reduced the release of vascular endothelial growth factor (VEGF) and tumor necrosis factor-alpha (TNF-α) [107]. VP12/14 and VP12/110 were shown to inhibit VEGF and TNF-α production by disrupting the interaction between HuR and TNF-α mRNA, thereby attenuating post-transcriptional regulation of inflammation.

### 6.2. Future Directions in Revealing Nutrient–RBP Interaction Mechanisms

The intersection of RBPs and nutrient metabolism represents a burgeoning frontier with transformative potential for understanding diet-related diseases. The current literature on RBPs faces several limitations that constrain the generalizability of findings. One major issue is the tissue-specific variability in RBP expression and function, as the same RBP may exert distinct regulatory roles depending on the cellular context, developmental stage, or metabolic state of a given tissue. Besides, much of the current understanding of RBP-mediated mechanisms is derived from animal models, which, while informative, may not fully recapitulate human-specific regulatory networks. The interspecies difference weakens the feasibility of translating preclinical findings into effective human therapies.

Future research will prioritize elucidating how RBPs dynamically sense and transduce nutrient signals to regulate metabolic pathways. One way is to identify specific RBPs that interact with functional nutrients such as vitamin C, folate, and prebiotics, and their roles in common metabolic diseases like obesity [108]. Also, the dysfunction of RNA-binding proteins is worth studying. They may act as a molecular bridge between transient hyperglycemia and sustained pathogenic gene expression, thereby contributing to the establishment and persistence of metabolic memory in diabetes. A critical focus will be deciphering the “nutrient-RBP-mRNA axis” in pathologies such as malnutrition and metabolic syndromes, particularly in understudied tissues like the gut–liver axis or adipose depots. CRISPR-Cas screens and chemical probes may identify RBPs that act as nutrient sensors, while engineered RBPs or synthetic RNA scaffolds could pioneer nutrient-guided therapeutics.

**Figure 4:**
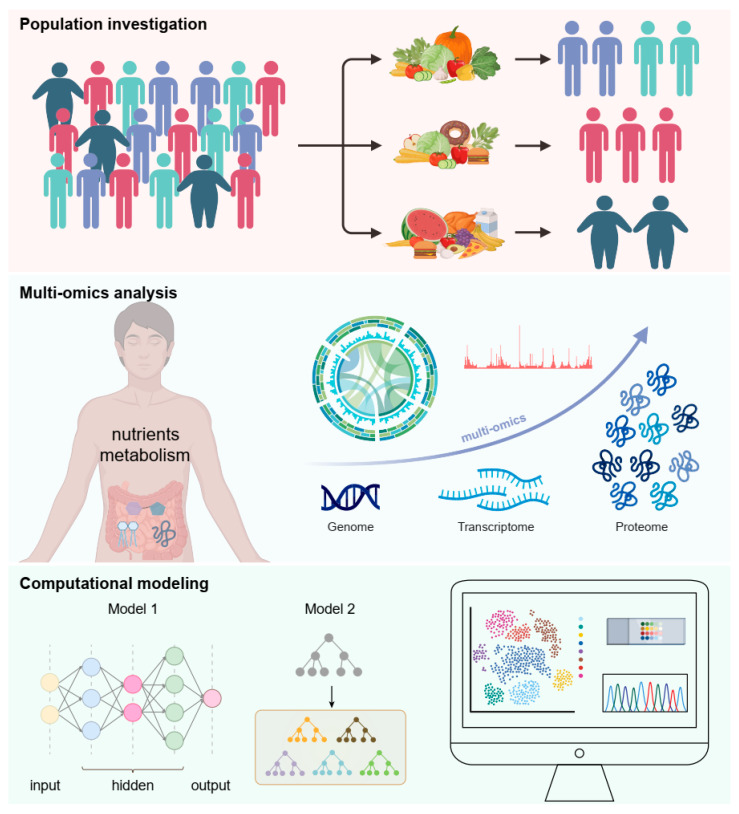
Future directions in RNA-binding protein research in nutrient metabolism.

Advanced techniques such as single-cell omics and spatial transcriptomics, combined with AI-driven integrative multi-omics platforms, will map tissue-specific RBP responses to dietary components like fatty acids, amino acids, and micronutrients. Machine Learning (ML) algorithms can identify subtle patterns in large-scale datasets, predicting how nutrient fluctuations alter RBP binding affinities or RNA regulon composition (Figure 4). For instance, deep learning models trained on CLIP-seq and metabolomic data may uncover novel “nutrient-RBP-mRNA axes” in pathologies such as obesity or malnutrition. CRISPR-Cas screens coupled with AI-powered drug discovery pipelines could accelerate the identification of nutrient-sensitive RBPs as therapeutic targets, while generative AI models might design synthetic small molecules to modulate RBP activity in a nutrient-dependent manner.

However, challenges such as inter-individual variability [109], delivery of RNA-targeted therapies, and regulatory hurdles must be carefully addressed before clinical implementation can be achieved. One possible solution would be employing federated ML frameworks to harmonize diverse clinical datasets while preserving privacy. Furthermore, reinforcement learning systems might optimize personalized dietary interventions by predicting how specific macronutrient ratios restore RBP-driven homeostasis in metabolic tissues. By merging RBP biology with precision nutrition, future studies could unlock diet-responsive RNA regulatory codes, paving the way for individualized therapies that exploit RBPs as dynamic sensors of nutritional status.

## 7. Conclusions

The emerging field of RBPs as regulators of nutrient metabolism represents a promising frontier in molecular and metabolic research. As our understanding of the intricate relationship between RBPs and metabolic transitions continues to evolve, several key areas warrant further investigation. A primary objective should be the systematic mapping of RBP–nutrient regulatory networks. Although significant progress has been made in identifying individual RBPs involved in metabolic regulation, a large number of interactions remain uncharacterized.

To address this gap, future studies should aim to identify novel RBPs implicated in metabolic control using high-throughput functional screening platforms. Advanced methodologies such as LACE-seq and CRIC-seq have demonstrated the ability to detect transient and context-specific RBP–RNA interactions. Expanding upon these tools and developing novel techniques—particularly those capable of capturing dynamic interactions in three-dimensional RNA structures—will be critical for uncovering mechanistic insights at a high resolution.

A more integrative approach, combining transcriptomic, proteomic, and metabolomic datasets, is essential for constructing a comprehensive framework of RBP-mediated metabolic regulation. Multi-omics integration will allow researchers to understand not only how RBPs affect gene expression and protein abundance, but also how these changes manifest at the level of cellular metabolism and nutrient flux.

With the development of sophisticated computational tools, AI technology is indispensable in identifying key regulators, screening therapeutic targets, and predicting functional outcomes of RBP perturbations. Such integrative and technologically advanced strategies will accelerate the discovery of novel regulatory mechanisms and facilitate the translation of RBP-based findings into therapeutic applications for metabolic diseases.

## 8. Methods

This review utilized PubMed, a comprehensive biomedical database maintained by the National Center for Biotechnology Information (NCBI), to identify relevant studies. The search covered publications from January 2000 to July 2025 to ensure a thorough analysis of historical and current evidence. Keywords include “RNA binding protein”, “obesity”, “NAFLD”, “diabetes”, “vitamin”, “iron”, “glucose”, “lipid”, “amino acid”, etc.

Given that this review is narrative in design, it may introduce subjectivity or selection bias compared to employing systematic screening protocols.
